# Hydro-marking Your Territory: Using a Novel Percutaneous Tagging System to Assist Pulmonary Nodule Identification During Video-assisted Thoracoscopic Surgery

**DOI:** 10.7759/cureus.3572

**Published:** 2018-11-12

**Authors:** Taylor S Harmon, Daniel Siragusa, James Cunningham, Travis E Meyer, Joanna Kee-Sampson, Jerry Matteo

**Affiliations:** 1 Interventional Radiology, University of Texas Medical Branch, Galveston, USA; 2 Interventional Radiology, University of Florida College of Medicine, Jacksonville, USA; 3 Radiology, University of Florida College of Medicine, Jacksonville, USA

**Keywords:** marker, hydrogel, lung cancer, mortality, pulmonary nodule, interventional radiology, percutaneous tagging system, vats, surgical resection, ct guidance

## Abstract

The management of lung cancer is highly dependent on surgical resection. There are different surgical managements that are utilized on a patient to patient basis. Small lung nodules are particularly difficult to resect and have a higher postoperative complication rate. Video-assisted thoracoscopic surgery is the preferred method of surgery over open thoracotomy, but requires the preoperative percutaneous placement of a marker to help the surgeon identify the nodule once conducting surgical resection. There are various methods to place percutaneous markers, but have reported complications. The following case will present the novel placement of a hydrogel tagging system on a small pulmonary nodule, demonstrating superiority to other methods of percutaneous marker placement.

## Introduction

Lung cancer remains the primary cause of mortality amongst all cancer types in both males and females in the United States [1]. Despite the continually large volume of lung cancer and subsequent death, the advancement of surgical procedures and detection methods has allowed for better patients outcomes and extended vitality. There is an overall three-year survival benefit for patients presenting with early stage metastatic disease who receive video-assisted thoracoscopic surgery (VATS) versus open thoracotomy [2]. In certain case scenarios however, visualizing small pulmonary nodules during VATS is difficult, with conversion to open thoracotomy approaching 54% in some series [3].

The current preoperative computed tomography (CT)-guided localization techniques reduce the rate of open conversion thoracotomy, but are also associated with pronounced limitations. Percutaneous marking of small nodules has been reported to have success when guiding for surgical resection, but has also resulted in complications such as pneumothorax, bleeding, and dislodgement [4]. The following case demonstrates a modified localization technique utilizing a hydrogel system that can be used to improve upon current percutaneous marking systems.

## Technical report

**Figure 1:**
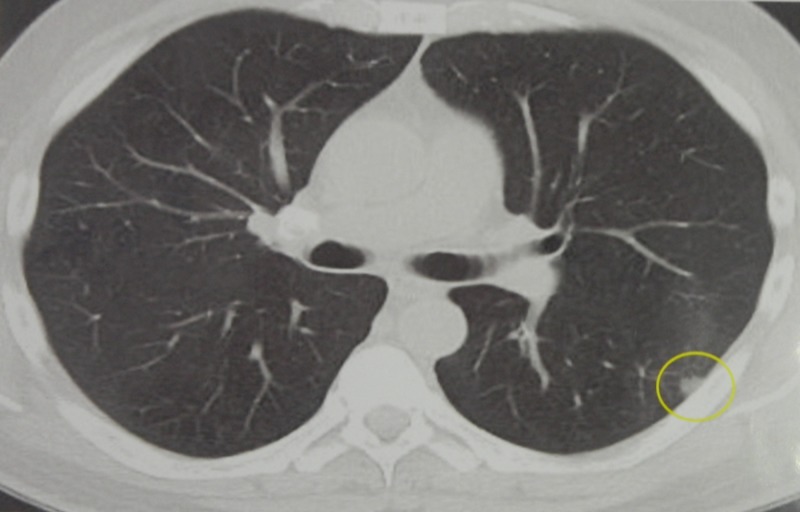
Axial Computed Tomography of a Small Pulmonary Nodule. An axial computed tomography study demonstrates a ground-glass nodular opacity in the left lower lobe (yellow circle).

A 60-year-old male with a past medical history of smoking had a CT of the chest that revealed a small six by six millimeter subpleural nodule in the left lower lobe (Figure 1).

**Figure 2:**
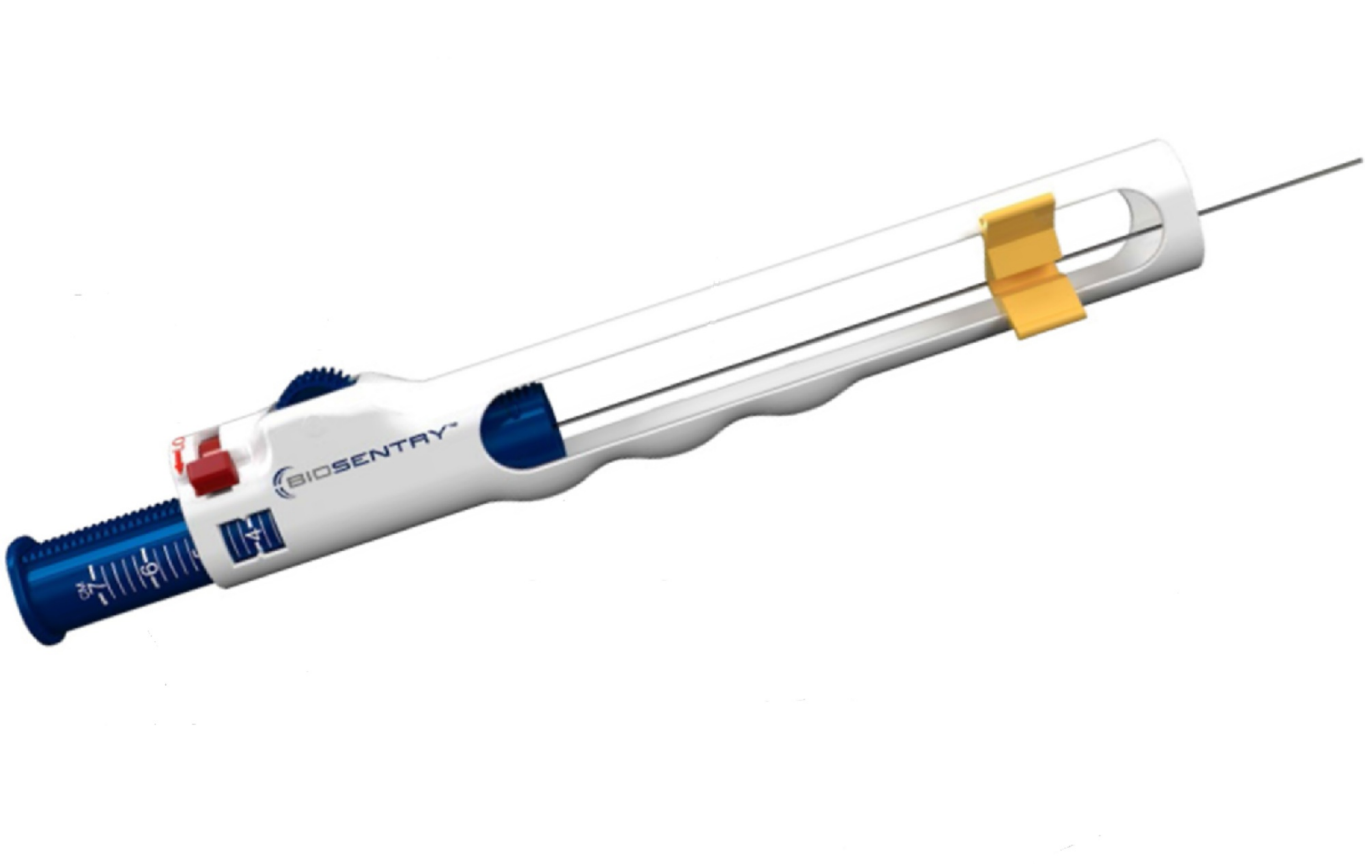
Biosentry® Deployment System. The figure represents the Biosentry® system that deployed the hydrogel placement marker within the small lung nodule.

The nodule had increased in size from a prior examination that was performed three years earlier, measuring two by three millimeters in diameter. The patient was subsequently offered a VATS resection of the subpleural nodule after confirming the growth of the nodule. CT guidance was utilized to preoperatively identify the pulmonary nodule. A 19-gauge coaxial needle was then directed within one centimeter of the nodule. Once the needle was positioned, a Biosentry® delivery device (AngioDynamics, Latham, New York) was loaded with a hydrogel plug that was activated with methylene blue instead of saline, in an attempt to visualize the plug during the VATS (Figure 2).

**Figure 3:**
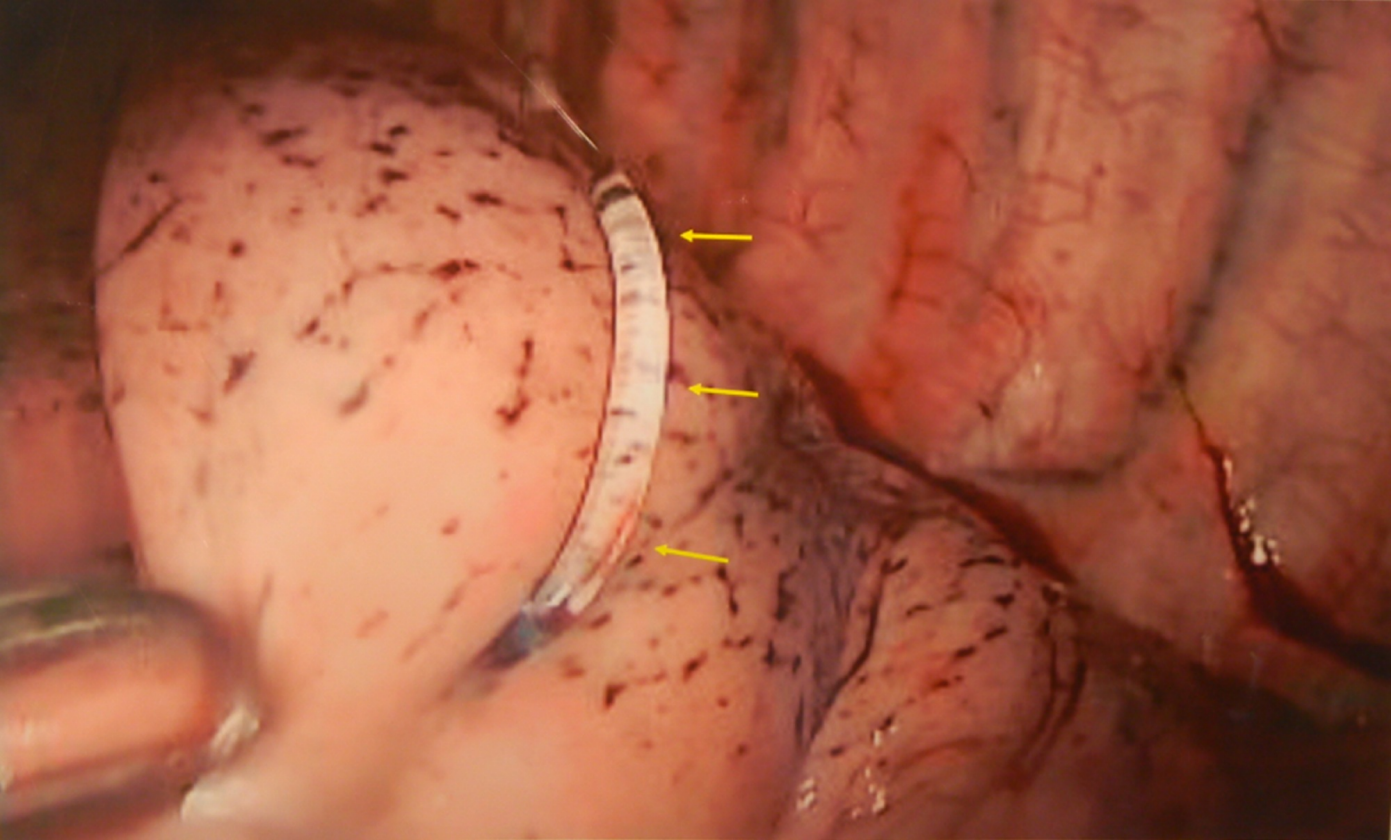
Intraoperative View of a Hydrogel Plug. An intraoperative view shows a hydrogel plug (yellow arrows) within the left lower lobe, as it passes through the small pulmonary nodule.

The hydrogel was then delivered through the needle so that a portion would remain in the pleural space and serve as a marker for eventual VATS resection (Figure 3).

Though the methylene blue was used to activate the hydrogel plug, the majority of the dye diffused into an adjacent lymph node. However, the hydrogel was still readily identifiable during the surgery. Following the percutaneous hydrogel deployment, the VATS wedge resection was successfully completed, and pathological samples revealed adenocarcinoma in situ.

## Discussion

Surgical interventions such as VATS provide a minimally invasive option for the resection of solitary pulmonary nodules. Moreover, VATS is associated with decreased length of hospital stay and postoperative pain, when compared to traditional open thoracotomy [5]. Though there is documented evidence supporting benefits of VATS over open thoracotomy, VATS-assisted wedge resection can be complicated in cases where lung nodules are particularly small. Traditional perioperative localization techniques to mark these small nodules have been used to guide subsequent surgical interventions, but have been known to cause certain complications including dye diffusion to untargeted tissues, wire dislodgement, bleeding, or lung trauma [6].

The hydrogel plug used in the preceding method presented by the case served as a readily identifiable marker for the target nodule, and did not cause any trauma or other associated complications to the lung parenchyma. Furthermore, it is in the nature of the hydrogel to swell when activated after deployment, leading to its secure placement. Unlike the placement of wires or other percutaneous markers, hydrogel does not dislodge with respiratory motion due to its chemical composition [7-9].

## Conclusions

Notably, VATS is readily used for the resection of small lung nodules over the more invasive open thoracotomy. Smaller lung nodules are usually preoperatively marked percutaneously under CT guidance in various ways to help surgeons who will perform VATS. However, traditional methods for percutaneous marking of small nodule lung cancer have been documented to have various complications. Hydrogel plug placement can safely localize small pulmonary nodules that may otherwise not be appreciated on VATS, without associated perioperative complications.
